# Genome Sequence of *Klebsiella pneumoniae* HSL4, a New Strain Isolated from Mangrove Sediment for Biosynthesis of 1,3-Propanediol

**DOI:** 10.1128/genomeA.00177-13

**Published:** 2013-05-09

**Authors:** Sheng Zhou, Lili Li, Jingguang Wei, Qiwei Qin

**Affiliations:** Key Laboratory of Marine Bio-resources Sustainable Utilization, South China Sea Institute of Oceanology, Chinese Academy of Sciences, Guangzhou, China

## Abstract

*Klebsiella pneumoniae* HSL4 is a 1,3-propanediol-producing bacterium strain isolated from mangrove sediment. We present here a 5,221,448-bp assembly of its genome sequence. Genome analysis revealed that it contains 10 coding sequences (CDSs) responsible for glycerol fermentation to 1,3-propanediol, 19 CDSs encoding glycerol utilization, and 140 CDSs related to its virulence.

## GENOME ANNOUNCEMENT

PDO (1,3-propanediol) has numerous applications in polymers, cosmetics, foods, lubricants, and medicines as a biofunctional organic compound (1). PDO is used mainly in the synthesis of polymers, such as the new polyester polytrimethylene terephthalate (PPT), which possesses better properties and greater stability than the polymers produced from 1,2-propanediol, butanediols, or ethylene glycol (2). In recent years, the production of PDO by microbial fermentation utilizing inexpensive, renewable crude glycerol as the substrate has become increasingly attractive (3, 4).

*Klebsiella pneumoniae* is one of the most widely investigated microorganisms that is capable of fermentation of glycerol to PDO with good yield, and the relevant metabolic pathways have been studied extensively (5). However, there are still some problems, such as the high concentration of by-product production and relatively low efficiency and low conversion from the substrate glycerol. The genome scale analysis has been proven useful for metabolic pathway analyses and metabolic engineering applications (6). Moreover, *K. pneumoniae* is a common opportunistic human pathogen, causing several diseases, such as pneumonia, bacteremia, and urinary tract infections (7). Therefore, it is necessary to reduce its pathogenicity by genetically modifying it for security so that it can serve as an industrial strain. This also requires that the genetic information and characteristics of *K. pneumoniae* be well understood. Genome sequencing and bioinformatics will be of great help in this regard.

*K. pneumoniae* HSL4 is a newly isolated strain with high efficiency in producing 1,3-PDO by fermentation of glycerol. Primary tests indicated that strain *K. pneumoniae* HSL4 could produce 80.08 g liter^-1^ 1,3-PDO, with a production intensity of 2.24 g liter^-1^ h^-1^ and conversion glycerol to PDO of 0.52 mol/mol. Here, we present the first draft genome sequence of *K. pneumoniae* HSL4 obtained using the Ion Torrent system, which was performed by the Guangzhou iGenomics Co., Ltd., Guangzhou, China, with a fragment library. The reads were assembled by using the GS *de novo* Assembler software from Roche. The draft genome sequence of strain HSL4 was annotated using the RAST annotation server (8). The G+C percentage was calculated using the genome sequence.

The draft genome sequence includes 5,221,448 bases and comprises 5,048 predicted coding sequences (CDSs) and 78 RNAs, with a G+C content of 57.5%, consisting of 211 large contigs (>200 bp in size). We have predicted 10 CDSs responsible for the fermentation of glycerol to 1,3-PDO, including the key genes of the 1,3-propanediol operon. Meanwhile, 19 CDSs responsible for glycerol utilization and 24 CDSs for 1,3-PDO utilization were annotated. These CDSs should be further investigated for elimination of by-product and increase in the productivity and conversion rate of glycerol. There are 140 CDSs that were annotated as the genes related to virulence, disease, and defense. About 107 of these CDSs were annotated as antibiotic- and toxic-compound resistance genes. Moreover, we annotated 43 CDSs related to the capsular and extracellular polysaccharides, which also correspond to virulence.

### Nucleotide sequence accession numbers.

This Whole-Genome Shotgun project has been deposited at DDBJ/EMBL/GenBank under the accession no. APFG00000000. The version described in this paper is the first version, accession no. APFG01000000.
